# Effects of milk, milk replacer, and milk replacer plus ethoxyquin on the growth performance, weaning stress, and the fecal microbiota of Holstein dairy calves

**DOI:** 10.3389/fmicb.2023.1113518

**Published:** 2023-03-13

**Authors:** Xiaoshi Wei, Jifu Zou, Yiwei Zhang, Jinyong Yang, Junhong Wang, Yanming Wang, Chong Wang

**Affiliations:** ^1^College of Animal Science and Technology, College of Veterinary Medicine, Zhejiang A&F University, Hangzhou, Zhejiang, China; ^2^Zhejiang Provincial Animal Husbandry Technology Promotion and Monitoring Station of Breeding Livestock and Poultry, Hangzhou, China; ^3^College of Animal Science, Zhejiang University, Hangzhou, China; ^4^Kemin (China) Technologies Co., Ltd., Zhuhai, China

**Keywords:** milk replacer, ethoxyquin, fecal fermentation, bacteria, body weight, weaning

## Abstract

The growth and health statuses of calves during the early stages of development have a significant effect on milk production during their first lactation period. Using appropriate milk replacers helps meet the long-term targets of dairy farmers. This study aimed to examine the effects of milk, milk replacer, and milk replacer plus ethoxyquin on growth performance, antioxidant status, immune function, and the gut microbiota of Holstein dairy calves. A total of 36 neonatal dairy calves were randomly divided into three groups and fed different diets: one group was fed milk, another group was fed milk replacer, and the third group was given milk replacer plus ethoxyquin. The supplementation with ethoxyquin was started on day 35 of the feeding period. The calves were weaned on day 45, and the experiment was conducted until day 49. The blood and fecal samples were collected at the end of the animal experiment. The results showed that milk replacers induced poor growth performance (body weight and average daily gain). However, milk replacer plus ethoxyquin aided in growth performance, enhanced the starter intake and blood antioxidative ability, and elevated the concentration of fecal valeric acid. Moreover, fecal fermentation and 16S rRNA analyses showed that milk replacer plus ethoxyquin altered the microbial composition (reducing *Alistipes and Ruminococcaceae* and increasing *Bacteroides and Alloprevotella*). Pearson's correlation assays showed that alterations in fecal microbiota strongly correlated with average daily gain and antioxidative ability. The results indicated the potential of milk replacer plus ethoxyquin in modulating the growth of dairy calves and in enhancing their ability to combat stress.

## Introduction

The growth and health statuses of calves during their first lactation period have a significant effect on milk production (Chester-Jones et al., 2017). Feeding *ad-lib* quantities of milk to dairy calves has been shown to cause higher growth rates at an early age (Khan et al., 2011; Iqbal et al., 2014). With the increasing demand for high production performance, milk replacer is an alternative that can boost growth performance but reduce the pre-weaning feeding cost.

Milk replacer is a type of artificial milk made of non-milk protein sources and is designed to meet the nutritional requirements of breast milk as per established standards. Compared to whole milk, the quality of milk replacer is not easily affected by external factors such as diet and season (Bernabucci et al., 2015; Toral et al., 2015). Calves fed milk replacers had a higher starter intake and longer-lasting effects on the rumen environment compared to those fed whole milk or pasteurized waste milk (Zhang et al., 2019). Exiting evidence also showed that milk replacers increase the survival rate of the Awassi lamb (Emsen et al., 2004). During the early stages of development, the gut microbiota is important to the host's health, as a stable gut bacterial community is a prerequisite for the host to perform normal physiological functions, metabolism, and immune functions (Gensollen et al., 2016; Li et al., 2021), while an imbalance may result in gastrointestinal diseases (Wang et al., 2019). Whether the gut bacterial community changes with the milk replacer is unknown.

Abrupt weaning, usually done at 6 weeks of age, is a source of stress for young animals and may lead to a reduction in body weight (de Passillé et al., 2011; Ungerfeld et al., 2011), diarrhea (Khan et al., 2007), and compromised intestinal barrier function (Li et al., 2018). Abrupt weaning makes young animals particularly vulnerable to infectious diseases as the immune system is not yet fully developed (Godbout and Glaser, 2006). Proper additives are a promising approach to protecting an animal from weaning stress (Kim et al., 2020; Mattioli et al., 2020). Ethoxyquin is widely used in animal feeds to protect against lipid peroxidation (Błaszczyk et al., 2013). Previous studies have demonstrated that feeding ethoxyquin can improve cows' lactation performance and antioxidant status, as well as partially mitigate the negative effects of feeding oxidized fat (Váquez-Añón et al., 2008; Boerman et al., 2014). Whether feeding ethoxyquin during the weaning period could help combat weaning stress is worth exploring.

We hypothesized that (1) a milk replacer could replace whole milk in dairy calves and (2) feeding ethoxyquin could mitigate negative effects during the weaning period. The objectives of this study were not only to determine the effects of milk, milk replacer, and milk replacer plus ethoxyquin on the growth performance and weaning stress of dairy calves but also to profile the changes in the gut microbiota.

## Materials and methods

### Experimental design and animal management

A total of 36 male Holstein calves were enrolled in this experiment. They were paired into 12 blocks based on their body weight and the date of their birth before being randomly assigned to one of three treatment groups within each block. The three treatments were control group (**C**, fed fresh milk), milk replacer group (**MR**, fed the milk replacer), and milk replacer plus ethoxyquin group (**MRE**, fed the milk replacer plus ethoxyquin). The ethoxyquin (Endox^®^5) was purchased from Kemin (China) Technologies Co. Ltd., Zhuhai. The dose of ethoxyquin used was 350 mg/kg of starter intake, and the feeding commenced on day 35.

The calves were given a total of 6 L of colostrum, with 4 L administered within the first hour after birth, and the remaining 2 L administered 5 h later. The calves were removed from their dam within 3 h of birth. The nutrient content of the milk replacer (Swot Technology Co., Ltd., Hangzhou) is presented in Table 1. Before feeding, the milk replacer was reconstituted with warm water (39°C) to 12.5% solids. The amount of milk or milk replacer fed to the calves was 12% v/w of their body weight, and the liquid feed was offered to them three times a day. They were fed from bottles at first but were then trained to drink from buckets. The day of birth was considered 1 day of age (**DOA**). After 38 days, the allowance of liquid feed was reduced by 50% each day, and the frequency of feeding was reduced to two times per day. At 42 days of age, the liquid feed was given one time per day. Weaning ended on day 45.

**Table 1:** The nutrient content of milk replacer.

**Items^a^**	**Contents**
Dry matter, %	94.3
Protein, %	22.4
Fat, %	12.5
Vitamin A, 10^4^ IU/kg	3.15
Vitamin D, 10^4^ IU/kg	0.65
Vitamin E, IU/kg	70
Calcium, %	0.75
Phosphorus, %	0.60

^a^The ingredient compositions are soy flour, whole milk powder, whey permeate, starch dextrin, calcium carbonate, dicalcium phosphate, lysine, methionine, threonine, vitamin premix, trace minerals premix, and additives.

**Figure 1:**
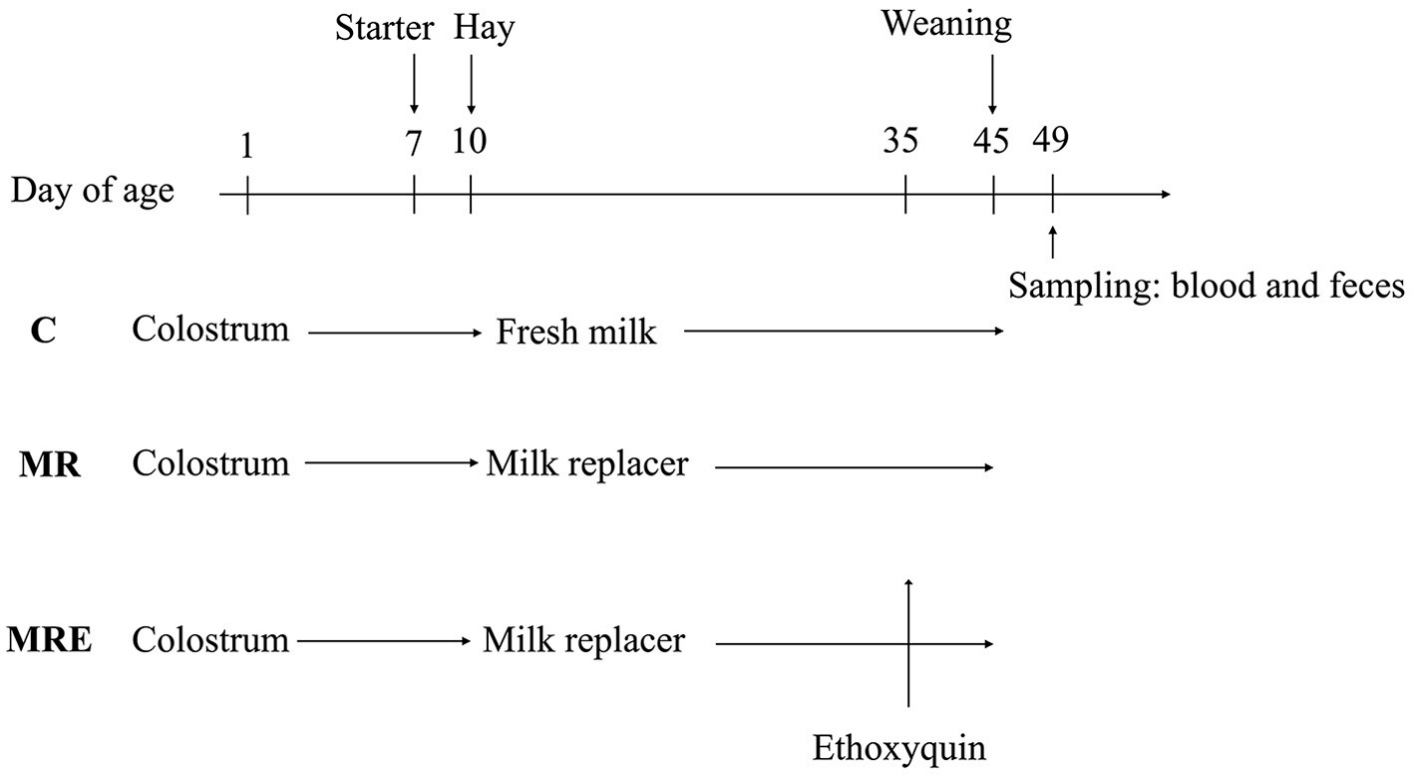
Overall timeline of the experimental protocol showing the change of treatments, starter, weaning and sample collection. C, calves fed fresh milk; MR, calves fed milk replacer; MRE, calves fed milk replacer plus ethoxyquin at 350 mg/kg group.

The pellets of a starter and alfalfa hay were offered *ad libitum* to the calves in individual buckets beginning at 7 DOA and 10 DOA, respectively. The amount of starter pellets intake was measured weekly to calculate the dose of ethoxyquin used. The chemical composition of the starter and alfalfa hay is shown in Table 2. All the calves were housed in individual hutches and managed similarly, with used sand as the bedding material. The sand was replaced one time a week to keep the bedding material clean. During the experiment, the windows of the hutches were opened for ventilation. The overall timeline of the experimental protocol is summarized and presented in Figure 1.

**Table 2:** The chemical composition of the feed.

**Items^a^, % of DM basis**	**Starter^b^**	**Alfalfa hey**
DM	89.9	89.4
CP	20.0	18.9
NDF	10.5	38.0
ADF	5.3	31.8
Ash	7.1	12.0
Ca	1.2	0.24
P	0.6	0.10

^a^DM, dry matter; CP, crude protein; NDF, neutral detergent fiber; ADF, acid detergent fiber; Ca, calcium; P, phosphorus.

^b^The ingredient compositions of the starter are corn, soybean meal, wheat bran, distillers dried grains with solubles, calcium carbonate, phosphate dicalcium, salt and premix.

### Intake and growth measures

The calves were weighed immediately after birth and weekly thereafter. The intake of starter pellets was recorded for each calf weekly.

### Blood sampling and analysis

Blood samples were collected at approximately 10 a.m. *via* the jugular venipuncture and divided into two tubes. One of the tubes contained K_2_-EDTA, and the samples were centrifuged at 3,500 g and 4°C for 15 min to obtain plasma. The sample in the other tube was allowed to clot at room temperature for 30 min to obtain serum. After centrifugation, all of the supernatants were then stored at −80°C for further analyses.

The plasma was analyzed for glucose, non-esterified fatty acids (**NEFA**), urea nitrogen (**BUN**), total protein (**TP**), and albumin (**ALB)** using commercial kits (Jiancheng Bioengineering Institute, Nanjing, China). The serum was used for analyzing the antioxidative status and immunity parameters. The total antioxidant capacity (**T-AOC**) was analyzed using the ferric antioxidant reducing power (FRAP) (Wang and Zuo, 2015). The activity of glutathione peroxidase (**GSH-PX**), as well as the malondialdehyde (**MDA**) concentration, were measured using commercial kits (Jiancheng Bioengineering Institute, Nanjing, China) following the manufacturer's instructions. The catalase (**CAT**) activity was determined using a commercial kit (Jiancheng Bioengineering Institute) based on the decomposition of hydrogen peroxide (H_2_O_2_). ELISA was used to determine the IgA, IgG, and IgM concentrations in serum (Cow IgA ELISA kit, catalog no. H108; Cow IgG ELISA kit, catalog no. H106; Cow IgM ELISA kit, catalog no. H109; and Jiancheng Bioengineering Institute, Nanjing, China).

### Feces collection and volatile fatty acid analysis

The feces were sampled in the last three consecutive days of the experiment so that the samples were represented every 3 h in a 24-h feeding cycle. After sampling, the fecal samples (about 200 g for each calf) were stored in liquid nitrogen immediately.

Before the analysis, all the fecal samples were pooled, mixed, and homogenized using a sterile slap homogenizer. Approximately 4 g of the samples was mixed in 4 mL of distilled water for volatile fatty acid (**VFA**) extraction and analysis. The concentrations and proportions of VFA (including acetic acid, propionic acid, butyric acid, valeric acid, isobutyric acid, isovaleric acid, and isoacids) were analyzed using gas chromatography (Agilent Technologies 7820A GC system, Santa Clara, USA) according to previously described methods (Li et al., 2019).

### Fecal microbiological analysis

#### DNA extraction and PCR amplification

Five samples from the treatment group were used for microbiological analysis. Approximately 1 g of the subsample was used for metagenomic DNA extraction. Microbial DNA was extracted from the fecal samples using the E.Z.N.A.^®^ soil DNA kit (Omega Bio-tek, Norcross, GA, U.S.) according to the manufacturer's protocols. The final DNA concentration and purification were determined using a NanoDrop 2,000 UV-vis spectrophotometer (Thermo Scientific, Wilmington, USA), and DNA quality was checked using 1% agarose gel electrophoresis. The V3-V4 hypervariable regions of the bacterial 16S rRNA were amplified with primers 338F (ACTCCTACGGGAGGCAGCAG) and 806R (GGACTACHVGGGTWTCTAAT) by a thermocycler PCR system (GeneAmp 9700, ABI, USA). The PCR reactions were conducted using the following program: 3 min of denaturation at 95°C, 28 cycles of 30 s at 95°C, 30 s for annealing at 55°C, and 45 s for elongation at 72°C, followed by a final extension at 72°C for 10 min. PCR reactions were performed in triplicates in a 20-μL mixture containing 4 μL of 5 × FastPfu Buffer, 2 μL of 2.5 mM dNTPs, 0.8 μL of each primer (5 μM), 0.4 μL of FastPfu Polymerase, and 10 ng of template DNA. The resulting PCR products were extracted from a 2% agarose gel and further purified using the AxyPrep DNA Gel Extraction Kit (Axygen Biosciences, Union City, CA, USA) and quantified using QuantiFluor™-ST (Promega, USA) according to the manufacturer's instructions.

#### Illumina MiSeq sequencing and processing

Purified amplicons were pooled in equimolar amounts and paired-end sequenced (2 × 300) on an Illumina MiSeq platform (Illumina, San Diego, USA) according to the standard protocols by Majorbio Bio-Pharm Technology Co. Ltd. (Shanghai, China). Raw FASTQ files were quality-filtered using Trimmomatic and merged using FLASH in accordance with the following criteria: (1) The reads were truncated at any site receiving an average quality score of < 20 over a 50-bp sliding window; (2) sequences whose overlap was longer than 10 bp were merged according to their overlap with a mismatch of no more than 2 bp; (3) sequences of each sample were separated according to barcodes (exactly matching), primers (allowing two nucleotide mismatches); and reads containing ambiguous bases were removed. Operational taxonomic units (OTUs) were clustered with a 97% similarity cutoff using UPARSE (version 7.1 http://drive5.com/uparse/) with a novel “greedy” algorithm that performs chimera filtering and OTU clustering simultaneously. The taxonomy of each 16S rRNA gene sequence was analyzed using the RDP Classifier algorithm (http://rdp.cme.msu.edu/) against the database using a confidence threshold of 70%.

The α diversity was analyzed using Mothur1.30.2 (https://www.mothur.org/wiki/Download_mothur). The β diversity analysis was based on the unweighted UniFrac distance and was performed using QIIME1.9.1. The microbiota composition at different levels was determined based on tax_summary and R package version 3.3.1, and the difference between the groups was analyzed using a one-way ANOVA and Tukey's test. The LDA effect size analysis (LEfSe) was conducted to screen differentially abundant bacterial taxa with an LDA score of >3.0.

### Statistical analysis

All the gathered data were analyzed using the MIXED procedure of SAS version 9.1 (SAS Institute Inc., Cary, NC). The repeated measures were used for the body weight (**BW**), average daily gain (**ADG**), and starter intake, and the model contained the effects of treatment, time, and the interaction of treatment and time. The initial BW was used for a covariate analysis. The linear model was used for fecal VFA concentrations and blood parameters. The results were expressed as least-squares means and standard errors of the mean. The correlations between fecal microbiota and performance, rumen fermentation, and blood parameters were calculated using Spearman's correlation coefficient. A heatmap diagram was drawn to visualize the data and identify the relationships between the variables. Statistical significance was determined for the treatment difference with a *P* ≤ 0.05, while a *P*-value of 0.05 < *P* ≤ 0.10 was considered indicative of a trend.

## Results

In total, five calves (1 in the C group, 3 in the MR group, and 1 in the MRE group) developed abomasal bloating. Consequently, they were removed from the experiment.

### BW, ADG, and starter intake of the calves

**Figure 2:**
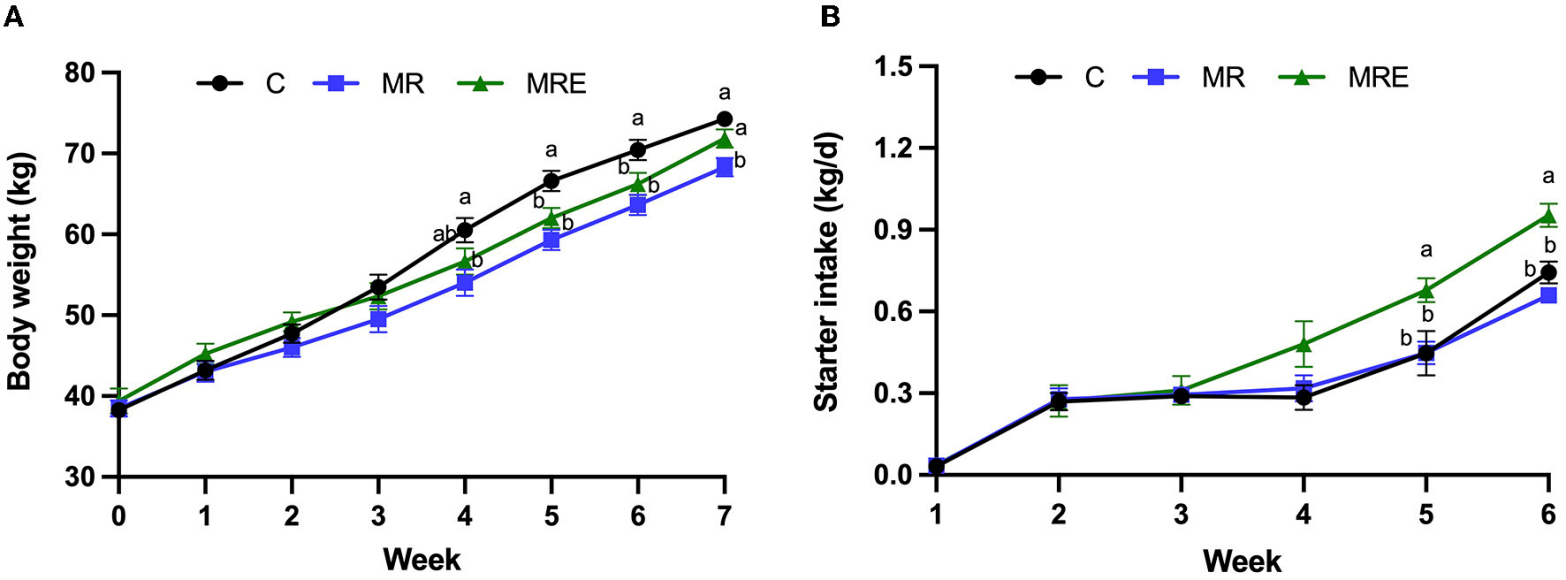
Growth performance of Holstein calves. C, calves fed fresh milk; MR, calves fed milk replacer; MRE, calves fed milk replacer plus ethoxyquin. **(A)** Body weight; **(B)** Starter pellets intake. Values were means ± SEM. ^ab^The mean values with different superscripts are different at *P* < 0.05. *N* = 11.9, and 11 in C, MR, and MRE, respectively.

The overall effects on BW, ADG, and starter intake are shown in Table 3. The results indicated a decrease in BW with MR treatment (*P* = 0.02), while MRE treatment led to an increase in starter intake (*P* < 0.01). Significant time effects were observed for both BW and starter intake (*P* < 0.01). The interaction between week × treatment was observed for both BW and starter intake (*P* < 0.01), and we detected a trend for the week × treatment interaction for ADG (*P* = 0.09). Compared to the C group, the calves in the MR group showed a decrease in BW at weeks 4 and 7, while both the MR and MRE groups showed a decrease in BW at weeks 5 and 6 (Figure 2, *P* < 0.05). In particular, the BW of the MR group at week 7 was significantly lower than that of both the C and MRE groups (*P* < 0.05). Additionally, at weeks 5 and 6, the starter intake was higher in the MRE group compared to the C and MR groups (*P* < 0.05).

**Table 3:** Effects of milk, milk replacer and milk replacer plus ethoxyquin on growth of Holstein calves (*N* = 11,9, and 11 in C, MR, and MRE, respectively).

**Items**	**Treatments** ^**1**^			**SEM**	***P*** **-value**		
	**C**	**MR**	**MRE**		**Wk**.	**T**	**Wk**. × **T**
Body weight, kg	57.1^a^	52.6^b^	56.3^a^	0.59	< 0.01	0.02	< 0.01
Average daily gain, kg/d	0.727	0.633	0.667	0.020	0.20	0.15	0.09
Starter intake, kg/d	0.362^b^	0.334^b^	0.461^a^	0.013	< 0.01	< 0.01	< 0.01

^1^C, control group; MR, milk replacer group; MRE, milk replacer plus ethoxyquin group.

^a, b^values in the same row with different small letter superscripts mean significant difference (*P* ≤ 0.05), while the same or no letter superscripts mean no significant difference (*P* > 0.05).

### Blood antioxidant ability and immunity parameters

The blood antioxidant ability and immunity parameters are shown in Table 4. The NEFA concentration was higher in the MRE group compared to the C and MR groups (*P* < 0.05), and the TP concentration tended to elevate (*P* = 0.06). There were no changes in the concentrations of glucose, BUN, or ALB. For antioxidant ability, the T-AOC was higher in the MRE group than in both the C and MR groups (*P* < 0.05). GSH-PX, CAT, and MDA also tended to increase (*P* = 0.07, 0.07, and 0.09, respectively). No difference was found in the immunity parameters (IgA, IgG, and IgM).

**Table 4:** Effects of milk, milk replacer and milk replacer plus ethoxyquin on blood metabolites of Holstein calves (*N* = 11.9, and 11 in C, MR, and MRE, respectively).

**Items^2^**	**Treatments** ^**1**^			**SEM**	***P*-value**
	**C**	**MR**	**MRE**		
**Blood parameters**					
Glucose, mmol/L	6.82	7.03	6.87	0.224	0.93
NEFA, mmol/L	0.127^b^	0.126^b^	0.172^a^	0.0085	0.03
BUN, mmol/L	6.78	5.28	5.50	0.418	0.30
TP, g/L	72.71	71.94	82.92	2.187	0.06
ALB, g/L	35.21	35.11	33.62	0.548	0.44
**Antioxidant ability**					
T-AOC, mmol/L	0.23^b^	0.24^b^	0.33^a^	0.016	0.01
GSH-PX, U/mL	115.26	126.43	157.52	7.857	0.07
CAT, U/mL	8.40	8.75	12.72	0.878	0.07
MDA, μmol/mL	2.55	3.06	4.04	0.285	0.09
**Immunity**					
IgA, mg/mL	2.81	3.52	3.39	0.155	0.14
IgG, mg/mL	12.59	13.40	12.14	0.393	0.44
IgM, mg/mL	4.07	4.73	4.28	0.284	0.65

^1^C, control group; MR, milk replacer group; MRE, milk replacer plus ethoxyquin group.

^2^NEFA, nonesterified fatty acid; BUN, blood urea nitrogen; TP= total protein; ALB, albumin; T-AOC, total antioxidant capacity; GSH-PX, glutathione peroxidase; SOD, superoxide dismutase; MDA, malonaldehyde; CAT, catalase; IgA, immunoglobulin A; IgG, immunoglobulin G; IgM, immunoglobulin M.

^a, b^values in the same row with different small letter superscripts mean significant difference (*P* ≤ 0.05), while the same or no letter superscripts mean no significant difference (*P* > 0.05).

### Fermentation parameters

As shown in Table 5, the concentration of acetic acid tended to decrease, and the concentration of valeric acid was increased in the MRE group compared to the C and MR groups (*P* = 0.08 and 0.05, respectively). Moreover, the ruminal isoacids (expressed as the percentage of total VFA) tended to increase (*P* = 0.07).

**Table 5:** Effects of milk, milk replacer and milk replacer plus ethoxyquin on fecal fermentation profiles of Holstein calves (*N* = 11,9, and 11 in C, MR, and MRE, respectively).

**Items^2^**	**Treatments** ^**1**^			**SEM**	***P*-value**
	**C**	**MR**	**MRE**		
**VFA**, μ**M**					
Acetic acid (A)	179.0	170.6	160.9	3.40	0.08
Propionic acid (P)	83.9	85.2	93.0	4.25	0.63
Isobutyric acid	8.02	11.18	10.72	0.65	0.12
Butyric acid	60.1	51.8	46.6	3.54	0.30
Isovaleric acid	8.40	13.58	14.86	1.30	0.16
Valeratic acid	8.94^b^	9.14^b^	11.18^a^	0.43	0.05
Isoacids	25.4	31.4	33.9	1.85	0.19
Total VFA	349.1	355.3	335.4	9.48	0.70
**VFA proportions, %**					
Acetic acid	51.2	50.3	45.2	1.40	0.16
Propionic acid	23.4	25.1	27.7	0.81	0.10
Isobutyric acid	2.64	3.30	3.16	0.21	0.59
Butyric acid	14.7	14.8	15.3	0.86	0.95
Isovaleric acid	2.93	3.97	4.13	0.38	0.51
Valerate	3.06	3.07	3.75	0.21	0.30
Isoacids	7.42	10.17	9.79	0.47	0.07
A:P ratio	2.18	2.12	1.69	0.11	0.13

^1^C, control group; MR, milk replacer group; MRE, milk replacer plus ethoxyquin group.

^2^VFA, volatile fatty acid.

^a, b^values in the same row with different small letter superscripts mean significant difference (*P* ≤ 0.05), while the same or no letter superscripts mean no significant difference (*P* > 0.05).

### Microbial community composition

**Figure 3:**
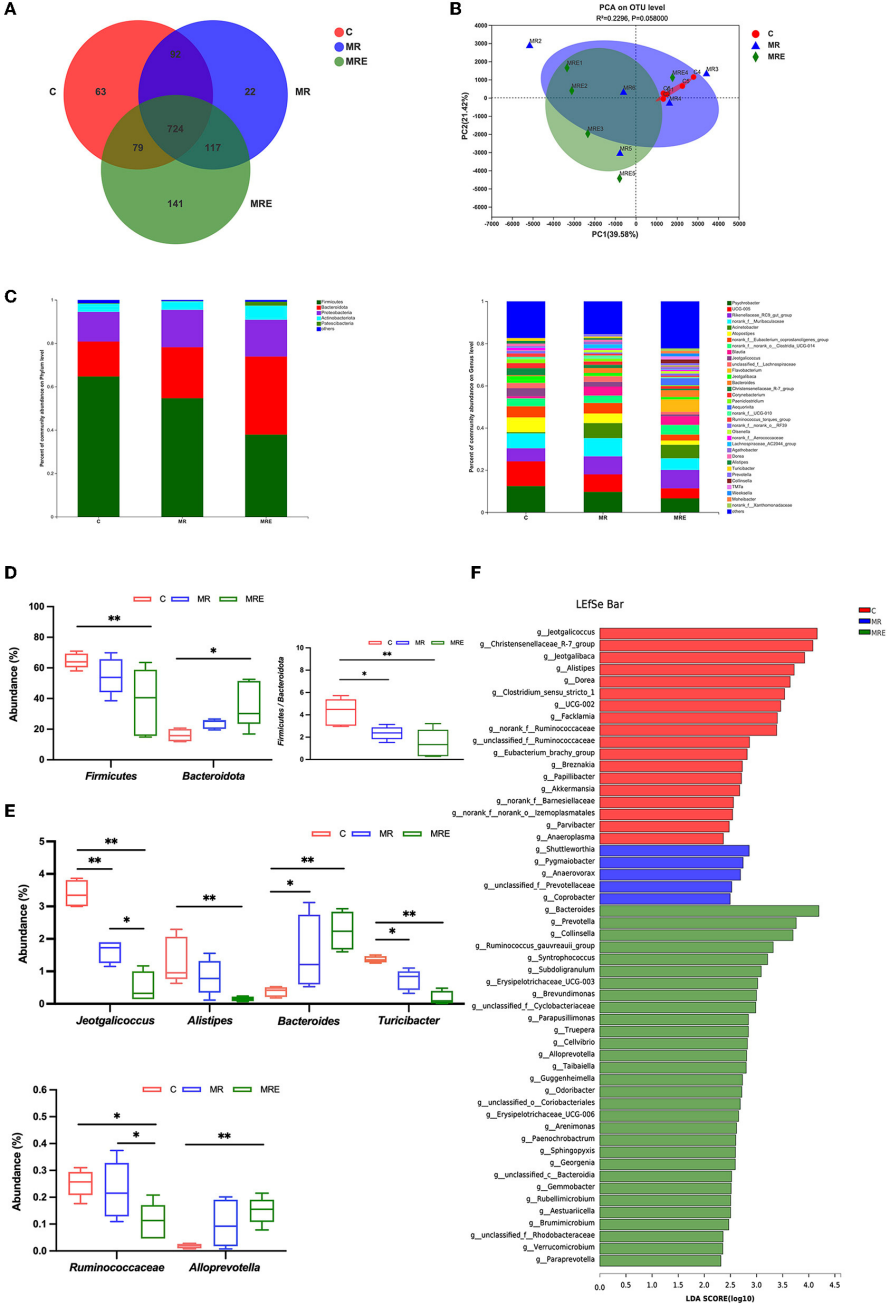
Analysis of the diversity, composition, and taxonomic biomarkers of fecal microbiota. C, calves fed fresh milk; MR, calves fed milk replacer; MRE, calves fed milk replacer plus ethoxyquin. *N* = 5 in each group. **(A)** Venn diagram presenting the operational taxonomic units (OTUs) from each group. **(B)** β diversity shown in a Principal component analysis (PCA) scatterplot. **(C)** A bar graph of microbial composition at both the phylum and genus levels. **(D)** A box plot of the significant phylum among groups. **(E)** A box plot of the significant genera among groups. **(F)** Histogram of Linear discriminant analysis (LDA) scores representing the taxonomic biomarkers by LDA effect size (LEfSe) analysis. LDA score (log10) >2 suggests the enriched taxa in cases. The data were analyzed by one-way ANOVA and Tukey's test. *P < 0.05, **P < 0.01.

The alterations in the fecal microbiota were investigated. The coverage for each sample was >99%, indicating sufficient sequencing depth to detect most of the fecal bacteria of the calves in this study. The total OTUs in the C, MR, and MRE groups were 958, 955, and 1,061, respectively (Figure 3A). Four indicators were used to reflect the microflora's richness (Chao, ACE) and diversity (Shannon, Simpson). As shown in Table 6, the richness was decreased in the MRE group compared to the C group (*P* < 0.05), with no difference in diversity. The β diversity was displayed in a PCA scatterplot and shown in Figure 3B, indicating a clear shift between the C and MRE groups.

**Table 6:** Effects of milk, milk replacer and milk replacer plus ethoxyquin on fecal bacterial diversity at OTU level of Holstein calves (*N* = 5).

**Items**	**Treatments** ^**1**^			**SEM**	***P*-value**
	**C**	**MR**	**MRE**		
Chao	715.8	694.0	577.3	27.2	0.08
Ace	724.4^a^	634.8^ab^	576.6^b^	24.2	0.02
Shannon index	4.44	4.25	4.27	0.07	0.53
Simpson	0.029	0.042	0.037	0.004	0.40
Coverage (%)	0.995^b^	0.995^b^	0.996^a^	0.000	0.02

^1^C, control group; MR, milk replacer group; MRE, milk replacer plus ethoxyquin group.

^a, b^Means within a row with different superscripts differ (*P* < 0.05).

The differences in microbial compositions at the phylum and genus levels are shown in Figure 3C. At the phylum level, the abundance of *Firmicutes* was decreased and that of *Bacteroidota* was increased in the MRE group compared to the C group (*P* < 0.05, Figure 3D), and the ratio of *Firmicutes* to *Bacteroidota* was lower in both the MR and MRE groups. The abundance of *Jeotgalicoccus* was significantly reduced in both the MR and MRE groups, and the abundance in the MRE group was the lowest (Figure 3E, *P* < 0.05). The abundance of *Alistipes* was found to be decreased, while the abundance of *Alloprevotella* was found to be increased in the MRE group compared to the C group (*P* < 0.01). The abundance of *Bacteroides* was increased in both the MR and MRE groups compared to the C group (*P* < 0.05 and *P* < 0.01, respectively). Moreover, the abundance of *Turicibacter* was decreased in both the MR and MRE groups (*P* < 0.05 and *P* < 0.01, respectively). The MRE significantly reduced the abundance of *Ruminococcaceae* compared with the C and MR groups (*P* < 0.05).

As shown in Figure 3F, the taxonomic biomarkers were *Jeotgalicoccus, Christensenellaceae_R-7_group, Jeotgalibaca, Alistipes, Dorea, Clostridium_sensu_stricto_1, Facklamia*, and *Ruminococcaceae* in the C group. In the MRE group, the predominant bacteria were *Bacteroides, Prevotella, Collinsella, Ruminococcus_gauvreauii_group, Syntrophococcus, Subdoligranulum*, and *Erysipelotrichaceae_UCG-003*. Additionally, no taxonomic biomarkers were found in the MR group under this condition.

### Relationship between bacterial and phenotypic variables

**Figure 4:**
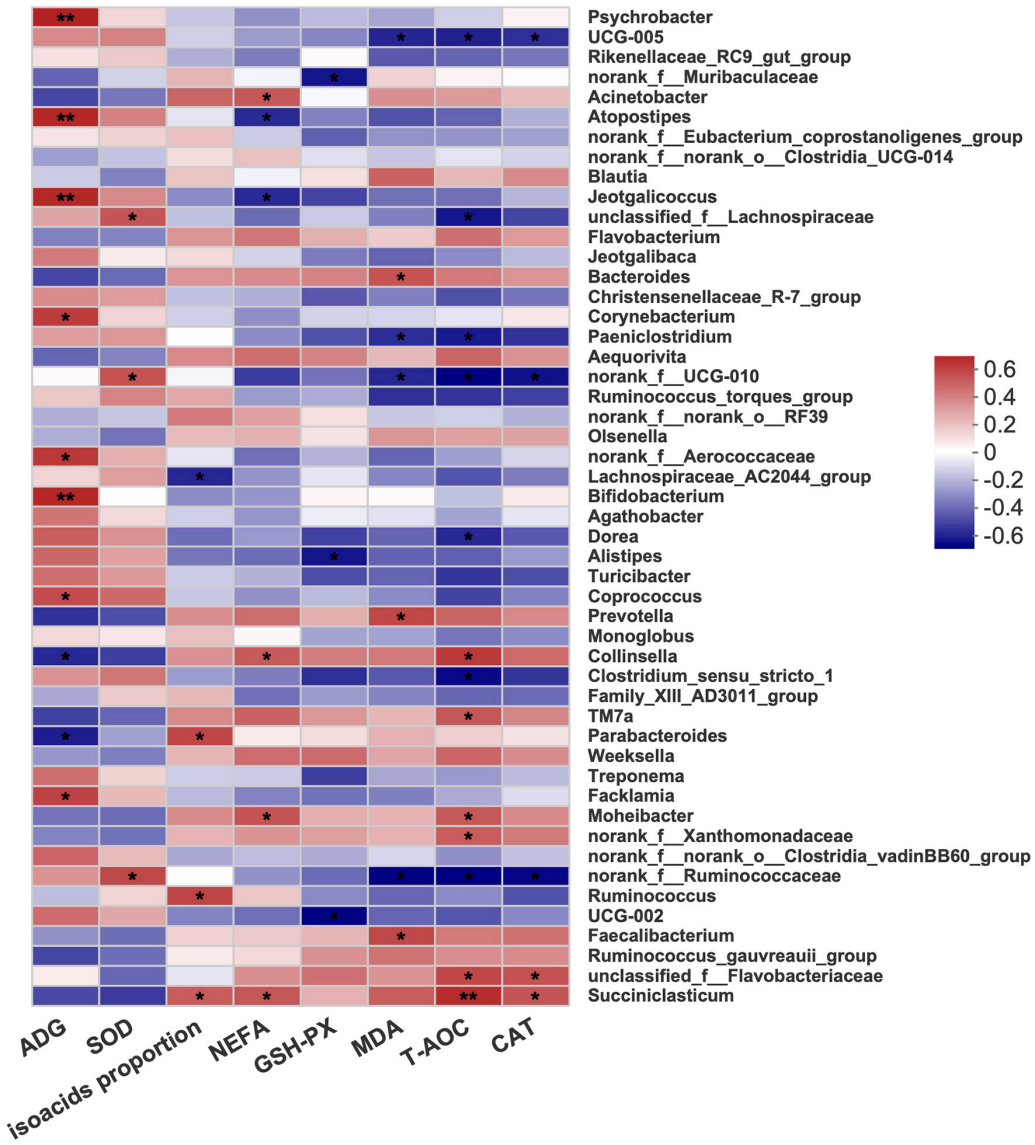
Heatmap diagram of correlations between fecal bacterial and performance and blood parameters at the genus level. Red was positively correlated and blue was negatively correlated. C, calves fed fresh milk; MR, calves fed milk replacer; MRE, calves fed milk replacer plus ethoxyquin. *N* = 5 in each group. Correlation significance *P*-value was indicated by “*”. *P < 0.05, **P < 0.01. ADG: average daily gain; SOD, superoxide dismutase; NEFA, nonesterified fatty acid; GSH-PX, glutathione peroxidase; MDA, malonaldehyde; T-AOC, total antioxidant capacity; CAT, catalase.

The correlations between fecal microbes, performance, and blood parameters were examined to further identify the underlying mechanisms. As shown in Figure 4, the concentration of MDA was positively correlated with *Bacteroides* and *Prevotella*. *Alistipes* showed a negative correlation with GSH-PX. Moreover, *Succiniclasticum* showed a positive correlation with CAT, T-AOC, NEFA, and isoacid proportions, while *Ruminococcus* was positively correlated with only isoacid proportions. *Lachnospiraceae* and *Ruminococcaceae* were positively linked with SOD. The results showed that the genera *Psychrobacter, Atopostipes, Jeotgalibaca, Corynebacterium, Aerococcaceae, Bifidobacterium, Coprococcus*, and *Facklamia* were positively related to the ADG.

## Discussion

This experiment evaluated the effects of milk, milk replacer, and ethoxyquin on growth performance, weaning stress, and fecal microbiota in dairy calves.

In this study, we found that the calves fed milk demonstrated superior growth performance and higher weaning weights.

The milk-fed calves showed better overall BW than those fed milk replacers (Zhang et al., 2019; Qadeer et al., 2021; Wang et al., 2022). The authors believe that this change might be due to the lower fat and protein contents and poor utilization of non-milk proteins. Moreover, milk might have better bioavailability of protein and energy along with minerals, enzymes, and growth factors (Lee et al., 2009). At the beginning and first few weeks, the BW of the dairy calves was not changed, while the milk replacer decreased the BW during weeks 4–7. A similar result was found in the study by Zhang et al. (2019), in which the BW of calves fed MR was significantly lower than that of those fed milk at 58 days of age. The nutrient contents of milk replacers were different in this study and that by Zhang et al. (2019). Previous studies demonstrated that the nutritional composition and the number of milk replacers provided significantly affect the BW of calves (Geiger et al., 2014; Chapman et al., 2016; Qadeer et al., 2021), suggesting that these factors are crucial.

The starter intake in the first 2 months was significantly associated with milk, fat, and protein production in the first lactation and lifetime production (Heinrichs and Heinrichs, 2011). Starter intake has been reported to be higher in milk-fed calves than those fed milk replacers, thus contributing to a higher growth rate (Qadeer et al., 2021). However, there was no difference in starter intake between the calves fed milk and those fed milk replacers in this study. The ethoxyquin helped to narrow the differences in BW and ADG between the calves fed milk and milk replacers. It might be due to the increased starter intake, as it could result in the compensation of nutrients to meet growth requirements. Feeding cows with ethoxyquin increased their DMI during mid and late lactation (Váquez-Añón et al., 2008).

Moreover, the higher consumption of starter intake may improve early rumen microbial development, leading to greater rumen capacity and metabolic activity (Anderson et al., 1987; Khan et al., 2011). Smith et al. (2003) reported that feeding antioxidants mixed with ethoxyquin improved the organic matter's digestibility. We speculated that the digestibility of DM and CP would be increased, and more research is needed to verify this fact.

In this study, the milk replacer did not change the blood metabolites, while the milk replacer plus ethoxyquin elevated the plasma concentration of NEFA. Feeding branched-chain VFA could decrease NEFA concentration in dairy cows (Liu et al., 2009), and a greater concentration of ruminal isovalerate might induce lower NEFA in calves (Zhang et al., 2019). Ethoxyquin increased the starter intake in this study, indicating that the rumen fermentation and bacterial community would be altered (Zhang et al., 2019). Moreover, the plasma NEFA is usually derived from fat stores as a response to energy mobilization; however, in this study, the BW and starter intake were increased in the MRE group compared to the MR group, indicating that an increase in NEFA by ethoxyquin would be due to the rumen metabolism but not the fat mobilization. A previous study demonstrated that antioxidants, such as vitamin E, can potentially prevent the “*trans*-10 shift” during biohydrogenation (Pottier et al., 2006). The ethoxyquin supplementation would preserve the oxidation of unsaturated fatty acids before absorption (Andrews et al., 2006) and increase the *cis-*18:1 in milk (Váquez-Añón et al., 2008), suggesting lower ruminal hydrogenation and trans-isomerization of 18:1 in some cases. The question of whether and how the ethoxyquin supplementation altered the rumen fermentation and microbiota profile will be evaluated in future studies.

Weaning is a potent stressor for dairy calves because of the extreme dietary shift, which induces elevated blood reactive oxygen species (ROS) production (Bordignon et al., 2019). Maintaining a balance between the defensive ability (enzymatic system and non-enzymatic antioxidants) and ROS production is important. Any disruption in this balance can lead to oxidative stress (Lykkesfeldt and Svendsen, 2007). The observed increase in antioxidant ability was expected, as ethoxyquin is one of the well-known feed antioxidant for both domestic animals and fish. It is widely used in animal feed due to its high antioxidant capacity and low production costs (Błaszczyk et al., 2013). Greater SOD and GSH-PX activities resulted from ethoxyquin supplementation in lactating primiparous cows (Váquez-Añón et al., 2008). As suggested by the authors, it is possible that ethoxyquin reduced a load of peroxides by removing reactive oxygen molecules, thereby relieving the endogenous antioxidant defense system. The elevated antioxidative ability we found in this study may have helped to mitigate the weaning impact on the calves.

In this study, the concentration of acetic acid was increased in the MR and MRE groups. The isoacids are the sum of isobutyric acid, isovaleric acid, and valeric acid. Branched-chain VFAs are markers of protein fermentation and are primarily derived from the fermentation of branched-chain amino acids such as valine and leucine (Smith and Macfarlane, 1998). In a study by Kumar et al. (2021), a higher concentration of milk replacer induced lower acetic acid and valeric acid proportions. Thus, we presumed that the higher concentrations of milk replacer-derived proteins and peptides would reach the intestine.

Despite the fecal fermentation, the microbiota was also evaluated in this study. The composition and balance of the microbiota are closely related to the nutritional and physiological functions of the host. Milk replacer has been shown to increase the diversity and richness of the microflora in the ileum (Wang et al., 2021). The results of Badman et al. (2019) proved that the differences in the nutritional composition of bovine milk replacers had a major impact on microbiota composition, diversity, and succession in pre-weaned dairy calves, further influencing the health of the gut and the whole animal. In this study, milk replacers did not affect the richness of the microflora, and milk replacer plus ethoxyquin decreased the richness with no difference in diversity. Combining the heatmap diagram and PCA, the results suggested that ethoxyquin might alter the composition of a milk replacer containing less widely utilized substrates for microbial fermentation.

Despite the genus mentioned above, *Firmicutes, Bacteroidetes, Proteobacteria*, and *Actinomycetes* usually account for more than 90% of gut microbes (Sankar et al., 2015). *Firmicutes*, an important indicator of intestinal microflora's composition, can be converted into short-chain fatty acids by fermenting polysaccharides to provide energy (Mariat et al., 2009). *Firmicutes* were also shown to promote energy absorption and fat deposition (Turnbaugh et al., 2006). We found that the abundance of *firmicutes* decreased in the MRE group compared to the C group, which suggested that the energy absorption and fat deposition would be lower, further favoring the elevated NEFA concentration. A previous study showed an increase in the abundance of *Bacteroidetes* in obese animals fed high-fiber diets (de Wit et al., 2012). The children who consumed a diet rich in fiber had higher proportions of *Bacteroidetes* and fewer *Firmicutes* than those fed a diet that included large amounts of protein, fat, sugar, and starch (De Filippo et al., 2010). However, we are unsure of why ethoxyquin can cause these changes.

Moreover, the ratio of *Firmicutes/Bacteroidetes* was significantly decreased. A previous meta-analysis revealed that a higher *Firmicutes*/*Bacteroidetes* ratio suggested more energy extraction from food by the microbiota (Suzuki and Worobey, 2014). Thus, we speculated that milk replacer and milk replacer plus ethoxyquin might alter the energy extraction from feed through microbiota, which could, in some cases, explain the decreased BW and ADG in this study. Moreover, we found that the abundance of *Bifidobacterium* was strongly correlated with ADG. *Bifidobacterium* is often among the first colonizers of gut environments (Malmuthuge et al., 2015) and is known to be beneficial to physiological conditions within the gut, aiding intestinal development and preventing intestinal dysbiosis (Hidalgo-Cantabrana et al., 2017).

The VFAs are important metabolites of the microbiota. Generally, acetic acid, propionic acid, and butyric acid are the primary fermentation products of *Ruminococcus albus, Prevotella ruminicola*, and *Butyrivibrio fibrisolvens*, respectively (Emerson and Weimer, 2017; Liu et al., 2017). The lower abundance of *Ruminococcaceae* in the MRE group tend to decrease the concentration of acetic acid. A previous study showed that the fermentation of branched-chain amino acids is mainly carried out by members of the genera *Clostridium, Peptostreptococcus*, and *Bacteroides* (Smith and Macfarlane, 1998), and the abundance of *Bacteroides* was found elevated in the calves fed milk replacer plus ethoxyquin in this study. Both *Ruminococcus albus* and *Butyrivibrio fibrisolvens* species are the main consumers of branched-chain VFAs (Feng, 2004). We also found that the abundance of *Ruminococcaceae* was lower in the MRE group, and the abundance of *Ruminococcus, Succiniciasticum*, and *Parabacteroides* were positively related to the isoacids proportion. Therefore, the decreased abundance of these bacteria might explain the greater proportion of isoacids. Moreover, previous studies demonstrated that isoacids could increase the number of cellulolytic bacteria (*fibrobacter succinogenes, Ruminococcus flavefaciens*, and so on) (Bryant and Doetsch, 1954; Dehority et al., 1967). Some *in vitro* studies have shown that isoacids can accelerate the degradation of DM and NDF (Soofi et al., 1982; Roman-Garcia et al., 2021). They tended to increase the proportion of isoacids, further suggesting that milk replacer and milk replacer plus ethoxyquin might influence the degradation and usage of feedstuffs. These might explain the increased starter intake in the MRE group.

The concentration of NEFA, the T-AOC, and the enzyme activity of CAT was positively correlated with *Succiniclasticum*, which is involved in the production of propionate (van Gylswyk, 1995). A lower abundance of *Ruminococcaceae* was detected in rats that were fed a high-fat diet (Zhao et al., 2017). The serum indicators of inflammation, such as TNF-α and IL-6, significantly increased. In this study, we also found that the antioxidative ability was negatively related to *Ruminococcaceae*, and the lower abundance of *Ruminococcaceae* further indicated a better health condition with ethoxyquin supplementation. Moreover, *Alistipes*, a potential opportunistic pathogen in diseases and highly relevant to dysbiosis and inflammation (Kong et al., 2019; Parker et al., 2020), was found to decrease and be negatively correlated with GSH-PX. These results also suggested that ethoxyquin improved the bacterial community.

## Conclusion

The results of this study suggest that milk replacers may not be sufficient to promote optimal growth performance in Holstein dairy calves during the early stages of life and that The addition of ethoxyquin could increase starter intake, thus narrowing the differences between the milk-fed and milk-replacer-fed calves. The results also suggested that milk replacer plus ethoxyquin enhanced the defensive ability and improved microbial composition to mitigate the negative effects of weaning.

## Data availability statement

The datasets presented in this study have been deposited in the NCBI repository, accession number PRJNA914621 (https://www.ncbi.nlm.nih.gov/bioproject/PRJNA914621).

## Ethics statement

The animal study was reviewed and approved by the Animal Care and Use Committee of Zhejiang A&F University, Zhejiang, China.

## Author contributions

XW and CW designed and supervised the study and revised the manuscript. XW, JZ, JY, and YW conducted the experiments. JZ, YZ, and JW performed the data analysis. XW drafted the manuscript. All authors read and approved the final version of the manuscript.
